# Sepsis is a preventable public health problem

**DOI:** 10.1186/s13054-018-2048-3

**Published:** 2018-05-06

**Authors:** Jordan A. Kempker, Henry E. Wang, Greg S. Martin

**Affiliations:** 1 Division of Pulmonary, Allergy, Critical Care and Sleep Medicine, Emory University School of Medicine, 49 Jesse Hill Jr Dr SE, Atlanta, GA 30303 USA; 2 Department of Emergency Medicine, University of Texas Health Science Center at Houston, 6431 Fannin Street, JJL 434, Houston, TX 77030 USA

**Keywords:** Sepsis, Infection control, Public health, Prevention, Epidemiology

## Abstract

There is a paradigm shift happening for sepsis. Sepsis is no longer solely conceptualized as problem of individual patients treated in emergency departments and intensive care units but also as one that is addressed as public health issue with population- and systems-based solutions. We offer a conceptual framework for sepsis as a public health problem by adapting the traditional model of primary, secondary, and tertiary prevention.

In 2017, the World Health Assembly and the World Health Organization (WHA/WHO) adopted a resolution prioritizing the reduction of the global burden of sepsis [1]. While clinical and scientific initiatives to date have primarily focused on the acute care of sepsis, the WHA/WHO resolution offers a major contrasting paradigm—that sepsis is a public health problem requiring population-based and systems-based solutions. Such a shift has happened before with myocardial infarction and stroke. While these were once viewed solely as acute, unpreventable conditions, today their prevention through the management of underlying chronic conditions is seen as one of the great public health achievements of the twentieth century [2]. With sepsis pursuing a similar trajectory, familiar—but important—questions arise. Can we reduce the attributable burden on society? What systematic interventions are most effective from a population perspective? And, ultimately, can we prevent sepsis?

Along these lines, we might conceptualize sepsis using the familiar model of primary, secondary, and tertiary prevention (Fig. 1). In this context, primary prevention refers to the prevention of infection or the sepsis event (i.e., onset of life-threatening organ dysfunction). Secondary prevention refers to the early recognition and treatment of sepsis. Tertiary prevention refers to in-hospital and post-hospital treatment to mitigate the long-term consequences of sepsis. While we know much about secondary prevention, work remains to be done in the realms of primary and tertiary prevention.

**Fig. 1:**
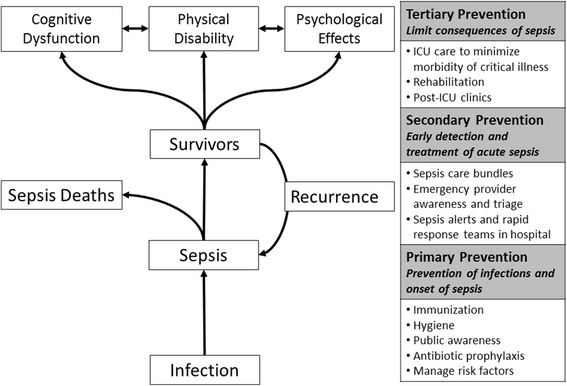
Preventive strategies along the sepsis chain of events. ICU intensive care unit

With regards to the primary prevention of sepsis, while sepsis may be new in the fields of public health, the prevention of infection has been a guiding principle for over 100 years. Today, systems continue to prevent sepsis via myriad strategies applied to different, constantly evolving scenarios. These include nationwide vaccine campaigns and outbreak management; hospital-wide policies on invasive medical devices, skin care, sterile techniques, hand hygiene, and quarantine; clinic-based surveillance and quarantine practices in high-risk specialty clinics such as those treating patients with cystic fibrosis; and individualized infection prophylaxis among those with immune compromise. These strategies have been immensely successful in reducing, and even eliminating, some of the burden of infectious diseases [2].

A second realm of primary prevention includes identifying the characteristics of high sepsis-risk individuals, similar to how the Framingham Study provided understanding of the risk factors for cardiovascular diseases. Early work with administrative healthcare data and contemporary cohorts such as the Reasons for Geographic and Racial Differences in Stroke cohort have provided major advancements to this knowledge gap for sepsis [3–5]. These and future works will be critical to help identify both modifiable risk factors and the risk groups in which to target future primary and secondary prevention strategies.

The notion of secondary prevention of sepsis has existing familiar foundations in clinical initiatives underscoring early recognition and treatment. Similar to myocardial infarction and stroke, sepsis has its “golden hours” where treatment with antibiotics are much more effective in reducing mortality [6, 7]. Today, the focus is on the effective implementation of systems-based processes to reduce sepsis mortality such as those promulgated by the Surviving Sepsis Campaign or those mandated by Centers for Medicare and Medicaid Services [8–10]. Future directions include measures that continue to move sepsis care earlier, with public awareness campaigns highlighting early recognition, call-ahead triage or early treatment by emergency medical services providers, and hospital-wide sepsis rapid response teams. Along these lines, the Centers for Disease Control and Prevention have initiated a Get Ahead of Sepsis campaign to increase public awareness of the early recognition and treatment of sepsis [11, 12].

Tertiary prevention refers to measures to limit the downstream effects of sepsis, optimizing the post-sepsis health trajectory. This trajectory includes sepsis recurrence, long-term mortality, and long-term morbidity across domains of psychological, cognitive, and physical function [13, 14]. While completing the picture of the total spectrum and societal burden of sepsis, the field of post-sepsis recovery is still in its early phases. There is a current need for health services and patient-centered outcomes research on strategies to reduce the long-term mortality and morbidity in sepsis. Until further evidence, the current strategies of tertiary prevention remain those universal to high-quality critical care: lung-protective ventilation, early awakening and mobility, early nutrition, reduction of device-associated complications, skin care, and early rehabilitation, among others. Future directions include examining interventions in the post-hospital care of sepsis survivors, which occurs across a variety of settings. These include long-term acute care, short-term rehabilitation, and nursing home facilities but may also involve acute care follow-up clinics.

While great public health benefits will result from the inclusion of sepsis into global health priorities of infection control, it is important to anticipate the challenges to this paradigm shift. First, sepsis is not a clearly defined disease entity but rather a nonspecific, clinical syndrome resulting from myriad combinations of pathogens, host responses, and organ dysfunctions. This creates conceptual and practical challenges in targeting public health strategies. Sepsis is difficult to diagnose, more difficult to surveil, and uniform strategies for quarantine do not apply. These challenges are compounded in resource-poor settings where the sepsis burden is unknown but undoubtedly great. A second challenge this paradigm shift faces is that the widespread adoption of early, empiric antibiotic treatment may have consequences that are antithetical to emerging principles in infection control. Specifically, these include the inappropriate use of antibiotics leading to depletion of a limited resource, an increase in adverse drug toxicities, and continued emergence of antimicrobial resistance.

The complexities of sepsis preclude any one-size-fits-all public health policy. The needs and high-yield strategies will differ between countries and healthcare systems and will depend on local pathogens, dominant host risk factors, strategies already in place, and resources. We think that these issues are precisely why sepsis belongs within the domain of a comprehensive global public health policy. A sepsis public health agenda that incorporates components of prevention, containment, early recognition, and treatment and balances policies within the context of downstream effects on public health is one that can achieve greater strides and, ultimately, the prevention of sepsis.
